# Fatty Acids and Frailty: A Mendelian Randomization Study

**DOI:** 10.3390/nu13103539

**Published:** 2021-10-09

**Authors:** Yasutake Tomata, Yunzhang Wang, Sara Hägg, Juulia Jylhävä

**Affiliations:** 1 Department of Medical Epidemiology and Biostatistics, Karolinska Institute, 171 77 Stockholm, Sweden; yunzhang.wang@ki.se (Y.W.); sara.hagg@ki.se (S.H.); juulia.jylhava@ki.se (J.J.); 2 Faculty of Health and Social Services, School of Nutrition and Dietetics, Kanagawa University of Human Services, Yokosuka 238-8522, Japan; 3 Gerontology Research Center (GEREC), Faculty of Social Sciences (Health Sciences), University of Tampere, 33014 Tampere, Finland

**Keywords:** fatty acids, frailty, Mendelian randomization

## Abstract

Background: Observational studies have suggested that fatty acids such as higher levels of n-3 polyunsaturated fatty acids (PUFAs) may prevent frailty. By using Mendelian randomization analysis, we examined the relationship between fatty acids and frailty. Methods: We used summary statistics data for single-nucleotide polymorphisms associated with plasma levels of saturated fatty acids (palmitic acid, stearic acid), mono-unsaturated fatty acids (MUFAs) (palmitoleic acid, oleic acid), n-6 PUFAs (linoleic acid, arachidonic acid), and n-3 PUFAs (alpha-linolenic acid, eicosapentaenoic acid, docosapentaenoic acid, docosahexaenoic acid), and the corresponding data for frailty index (FI) in 356,432 individuals in the UK Biobank. Results: Although there were no robust associations on the MUFAs or the PUFAs, genetically predicted higher plasma stearic acid level (one of saturated fatty acids) was statistically significantly associated with higher FI (β = 0.178; 95% confidence interval = −0.050 to 0.307; *p* = 0.007). Such a relationship was also observed in a multivariate MR (β = 0.361; 95% confidence interval = 0.155 to 0.567; *p* = 0.001). Genetically predicted higher palmitic acid was also significantly associated with higher FI (β = 0.288; 95% confidence interval = 0.128 to 0.447; *p* < 0.001) in the multivariate MR analysis. Conclusions: The present MR study implies that saturated fatty acids, especially stearic acid, is a risk factor of frailty.

## 1. Introduction

Fatty acids are one of the nutritional components that contribute to healthy aging [1]. For example, n-3 polyunsaturated fatty acids (PUFAs) such as docosahexaenoic acid (DHA) and eicosapentaenoic acid (EPA) are known to reduce inflammation, suggesting a beneficial role in various therapeutic areas [2]. There is, however, sufficient evidence for an anti-inflammatory effect on aging only for n-3 PUFA intake [3]. As for n-3 PUFAs, evidence on benefits for cognitive health and age- and disease-related decline in muscle mass has been presented [2,4,5,6].

There is some evidence from observational studies showing an association between fatty acids and frailty [7]. The overall results suggest that n-3 PUFAs are inversely associated with the risk of frailty [8]. The observational study using U.S. nationally representative data (the National Health and Nutrition Examination Survey) also showed that low n-3 PUFA intake (DHA and EPA) was associated with a higher value of frailty index (FI) in participants aged ≥20 years. In addition, mono-unsaturated fatty acids (MUFAs) were also suggested as preventive factors for frailty [9]. On the other hand, higher saturated fatty acid intake was associated with higher risk of both frailty and mortality even after considering the degree of nutritional deficits [10]. Thus, in conclusion, various types of fatty acids may contribute to frailty.

However, observational studies may have methodological problems, such as confounding or reverse causality. For example, a pooled analysis with data from cohort studies showed that higher n-3 PUFA levels were significantly associated with a lower risk of cardiovascular disease-related deaths [11]. However, a meta-analysis of randomized controlled trials showed that n-3 PUFA had no significant association with cardiovascular disease [12]. The discrepancies in these results may be explained by residual confounding or reverse causality in observational studies [11]. An RCT is more suitable to examine causal relationships without confounding concerns [12]. However, regarding frailty, no randomized controlled trial (RCT) has yet been performed to analyze the effect of fatty acids.

To overcome the problem of confounding and reverse causality in observational studies, Mendelian randomization (MR) is becoming widespread for assessing whether the association is consistent with a causal hypothesis. An MR study is a type of instrumental variable analysis where genetic variants (single-nucleotide polymorphisms; SNPs) are applied as instrumental variables for the exposure. An MR study is often described as a “natural RCT” because random allocation of alleles during meiosis is conceptually similar to RCT design. Therefore, an MR study would provide more robust evidence regarding the causal relationship between fatty acids and frailty than observational studies. However, to our knowledge, no MR study has yet investigated the associations between fatty acids and frailty, although there have been several MR studies on the relationship between fatty acids and cardiovascular disease [13,14,15,16,17].

The aim of the present MR study was to examine the associations between fatty acids and frailty.

## 2. Materials and Methods

### 2.1. Study Design

Two-sample MR analyses using summary statistics of a genome-wide association study (GWAS) were conducted. As exposure measures, we examined ten types of plasma fatty acids: two types of saturated fatty acids (palmitic acid, stearic acid), two types of MUFAs (palmitoleic acid, oleic acid), two types of n-6 PUFAs (linoleic acid, arachidonic acid), and four types of n-3 PUFAs (α-linolenic acid, eicosapentaenoic acid, docosapentaenoic acid, docosahexaenoic acid) [18]. As an outcome measure of frailty, the Rockwood FI was used [19].

### 2.2. Data Sources

For estimating the genotype–FI associations, we analyzed individual-level data from participants enrolled in the UK Biobank [20]. The UK Biobank is a population-based cohort study on individuals aged from 40 to 69 years recruited from the UK National Health Service registers. The UK Biobank includes data on genetic and environmental factors across the UK collected from 2006 to 2010 (https://www.ukbiobank.ac.uk/ [accecced on 3 March 2021]). Among the 500,336 participants who had information about FI available [19], we included 356,432 participants who were (1) genotyped, (2) of European ancestry, (3) had complete information on variables included in the FI, and (4) not randomly dropped with a kinship threshold of 0.1768 for considering relatedness. The analytical sample comprised 189,949 women (53.3%) and 166,483 men (46.7%), with a mean (SD) age of 56.7 (8.0) years.

We used summary statistics data from the Cohorts for Heart and Aging Research in Genomic Epidemiology (CHARGE) consortium to examine the association between genetic variants and plasma levels of fatty acids [21,22,23]. The summary statistics were based on a meta-analysis of GWAS of individuals of European ancestry (*n * =  8961 individuals for saturated fatty acids or mono-unsaturated fatty acids, *n*  =  8631 individuals for n-6 PUFAs, and *n*  =  8866 individuals for n-3 PUFAs). Age ranged from 21 to 102 years [23].

### 2.3. Selection of Instrumental Variables

We selected SNPs associated with the plasma fatty acids (*p* < 5 × 10^−8^) in the previous GWAS meta-analysis in individuals of European ancestry (Table 1) [21,22,23]. As one SNP (rs11190604) was not available in the UK Biobank data, we used an overlapping proxy SNP (rs7099965) in high-linkage disequilibrium (r^2^ = 1) identified using the LDproxy search on the online platform LDlink (https://ldlink.nci.nih.gov/ [accecced on 3 March 2021]). These SNPs have been used as instrumental variables for plasma fatty acids in previous MR studies [18] and were not considered as weak instrumental variables (F statistic ≥ 10) [24].

To consider the possibility that palindromic SNPs alter the results associated with frailty in several fatty acids (stearic acid, linoleic acid, eicosapentaenoic acid), we also used proxy SNPs in high-linkage disequilibrium (r^2^ > 0.90) instead of three SNPs; rs11119805, rs10740118, and rs3798713 (Table 2).

### 2.4. Frailty

The Rockwood FI, based on the accumulation of deficits model, was used as an outcome measure of frailty. The FI is a continuous measure with a high sensitivity also at the lower end of the frailty continuum. According to the principles of this model [25], the FI value for each individual was calculated as the number of deficits present divided by the total number of 49 deficits as described in a previous study [19]. In the present study, the FI value was expressed as a percentage (%). For example, an individual having 10 deficits has an FI of 10/49 = 0.204 (20.4%).

### 2.5. Statistical Analysis

We estimated the association of each genetic variant with the continuous FI using linear regression analyses to obtain summary statistics for the two-sample MR analyses. All the regression analyses were adjusted for age (continuous variable) and sex using SAS version 9.4 (SAS Inc., Cary, NC, USA).

Two-sample MR analyses were performed to calculate the coefficients (linear association) and 95% confidence intervals (CIs) for FI. All MR analyses were performed by using the “MendelianRandomization” package for R version 3.4.3 (The R Foundation for Statistical Computing). We primarily used the inverse variance weighted method with fixed-effect standard errors [26]. The inverse variance weighted method assumes that all variants are valid instrumental variables.

In addition, we also conducted sensitivity analyses using the MR-Egger method for exposures that used three or more SNPs as instrumental variables (i.e., stearic acid, palmitoleic acid, linoleic acid, docosapentaenoic acid). The MR-Egger method estimates the effect size by adjusting for horizontal pleiotropy (i.e., the genetic variants have effects on the outcome through other paths than via the exposure of interest) [26]. Pleiotropy was assessed using the MR-Egger intercept test, which assumes that the intercept should be zero if the genotype–exposure association is zero.

## 3. Results

### 3.1. Summary Statistics

By using the linear regression analyses, we obtained summary statistics for the associations between the genetic variants and FI that were needed to conduct the MR analysis (Table 1 and Table 2).

### 3.2. Main Results

The results of the MR analyses based on the inverse variance weighted method are shown in Table 3.

In the non-PUFAs, genetically predicted plasma stearic acid level was statistically significantly associated with higher value of FI in both the MR (β = 0.178; 95%CI = −0.050 to 0.307; *p =* 0.007) and the multivariate MR (β = 0.361; 95%CI = 0.155 to 0.567; *p =* 0.001). In the multivariate MR analysis, palmitic acid was also significantly associated with higher FI (β = 0.288; 95%CI = 0.128 to 0.447; *p* < 0.001).

Of the PUFAs, none of the genetically predicted plasma PUFA levels were statistically significantly associated with FI in the multivariate MR.

There was no evidence for pleiotropy based on the MR-Egger analyses in all four types of fatty acids—stearic acid, palmitoleic acid, linoleic acid, and docosapentaenoic acid (P-intercept > 0.05).

### 3.3. Sensitivity Analysis

The results of the sensitivity MR analyses using proxy SNPs are shown in Table 4. Genetically predicted plasma stearic acid level was statistically significantly associated with higher FI in both the MR (β = 0.177; 95%CI = 0.049 to 0.305; *p =* 0.007) and the multivariate MR (β = 0.360; 95%CI = 0.154 to 0.566; *p =* 0.001).

## 4. Discussion

The present study is an MR study that examines the relationship between genetically predicted levels of fatty acids and frailty. Our results showed that two major saturated fatty acids (palmitic acid, stearic acid) were statistically significantly associated with higher FI even after using the multivariate MR. However, there were no robust associations on the major types of MUFAs or PUFAs (e.g., oleic acid, eicosapentaenoic acid, docosahexaenoic acid) in the multivariate MR.

In the results of the present study, higher stearic acid was consistently associated with higher FI in both the MR and the multivariate MR (Table 3). Furthermore, the MR-Egger analysis suggested no evidence that the result of stearic acid-induced pleiotropy. The results of the multivariate MR showed that palmitic acid was also associated with a higher FI. Thus, our results support the hypothesis that higher levels of saturated fatty acids are risk factors for frailty. Stearic acid and palmitic acid are the major dietary saturated fatty acids typically reflected by adherence to a Western diet including a relatively high amount of animal fat [27]. A previous observational study reported that a higher percentage of saturated fatty acid intake was associated with a higher risk of both frailty and mortality, even after considering the degree of nutritional deficits [10]. A population-based cohort study suggested that the animal protein intake was significantly associated with a higher FI in adults (aged ≥55 years) of normal body weight, and saturated fatty acids tended to be associated with a higher FI in the same population [28]. The observational study revealed that a Westernized-type dietary pattern (characterized by the highest saturated fats) was strongly associated with a higher frailty prevalence, whereas Mediterranean-type dietary pattern (characterized by relatively low intake of meat, egg, fish, and dairy products) was inversely associated with the prevalence of frailty [29]. Another cohort study also revealed that a Westernized dietary pattern (characterized by a high intake of refined bread, whole dairy products, and red and processed meat, as well as low consumption of fruit and vegetables) tended to be associated with a higher risk of frailty [30]. Meta-analyses of observational studies showed associations between saturated fatty acids intake and a high risk of coronary heart disease mortality [31], bone fracture [32], or cognitive disorders [33]. Indeed, a MR study based on UK biobank data revealed that of the major fatty acids, stearic acid was associated with a high risk of cardiovascular diseases such as aortic valve stenosis and venous thrombosis [13]. Another MR study based on UK biobank data also revealed that stearic acid was significantly associated with lower bone mineral density and a higher risk of fracture [34]. Furthermore, MR study-based DIAGRAM consortium including UK biobank data showed that stearic acid was significantly associated with a higher risk of type 2 diabetes. Hence, it would be expected that reduction in the dietary intake of saturated fatty acids contributes to the prevention of frailty via multiple traits. However, based on the current evidence, it is unclear whether dietary saturated fatty acids should be substituted by MUFAs or PUFAs. For example, a previous cohort study reported that substitution of saturated fatty acids (palmitic acid and stearic acid) with plant proteins resulted in a reduction of myocardial infarction risk, but not substitution with MUFAs or PUFAs [35].

However, it is open for discussion whether stearic acid is a specific risk factor of most frailty components or not. For example, a previous cohort study reported that plasma stearic acid was associated with a higher risk of colorectal cancer, although dietary palmitic acid was inversely associated with colorectal cancer [36]. A previous MR study also supported that stearic acid (but not palmitic acid) increases colorectal cancer risk [18]. However, one previous cohort study reported that higher concentrations of plasma phospholipid stearic acid were associated with a lower risk of all-cause mortality, although palmitic acid was associated with a higher risk of total mortality [37]. For example, as for cardiometabolic risk factors, substituting palmitic acid with stearic acid lowers LDL-cholesterol concentrations [38]. Thus, further studies are needed to verify whether only stearic acid is a risk factor among the saturated fatty acids.

As one of major methodological issues about bias towards the null in MR studies, weak instrument bias is often suggested. Although we used only 1 to 3 SNPs as instrumental variables for each PUFA, the SNPs fulfilled the criterion as not being weak instrumental variables (F statistic > 10). However, the change in coefficients and 95%CIs between the MR and the multivariate MR (Table 3) may not be interpreted as robust estimates.

Our study had several strengths. First, to our knowledge, the present study is the first MR study to examine the associations between fatty acids and frailty. This approach (MR study) is less prone to confounding compared to conventional observational studies. Second, we conducted several sensitivity analyses to check robustness of the main results such as the multivariate MR. The present study has also several limitations. First, we only used 1 to 3 SNPs as instrumental variables for each PUFA. Therefore, only four types of fatty acids (stearic acid, palmitoleic acid, linoleic acid, docosapentaenoic acid) could be used for pleiotropy test by the MR-Egger intercept test. Second, because shared SNPs were used among the fatty acids (e.g., rs174547 was used for LA, AA, ALA, and DPA), identifying the effects for individual fatty acids was difficult. Although we thereby conducted the multivariate MR to overcome this problem, these results would not be robust estimates as stated before. Third, because our analysis was restricted to populations of European ancestry, the present findings may not apply to non-European populations.

## 5. Conclusions

The present MR study suggested that saturated fatty acids (especially stearic acid) is a risk factor of frailty. Nevertheless, future studies may need to consider the balance of fatty acid composition rather than the effect of a single component of fatty acid.

## Figures and Tables

**Table 1:** Summary statistics of fatty acids-raising genetic variants.

			Effect Allele	Other Allele	Fatty Acids ^a^				Frailty Index ^b^		
Traits	SNPs	Chr			EAF	*β*	SE	*p*	*β*	SE	*p*
PA, 16:0	rs2391388	1	C	A	0.45	0.178	0.027	2.72 × 10^−11^	−0.011	0.017	0.518
SA, 18:0	rs6675668	1	G	T	0.51	0.165	0.019	2.16 × 10^−18^	0.016	0.017	0.360
	rs11119805	1	T	A	0.88	0.168	0.028	2.80 × 10^−9^	−0.023	0.026	0.371
	rs102275	11	T	C	0.68	0.180	0.019	1.33 × 10^−20^	0.070	0.018	1.00 × 10^−4^
POA, 16:1n−7	rs780093	2	T	C	0.41	0.020	0.003	9.80 × 10^−10^	0.045	0.018	0.011
	rs6722456	2	G	A	0.98	0.048	0.009	4.12 × 10^−8^	−0.028	0.058	0.629
	rs603424	10	G	A	0.81	0.033	0.004	5.69 × 10^−15^	−0.049	0.023	0.033
	rs7099965	10	G	A	0.22	0.023	0.004	6.96 × 10^−9^	−0.041	0.021	0.048
	rs102275	11	C	T	0.33	0.024	0.003	6.60 × 10^−13^	−0.070	0.018	1.00 × 10^−4^
OA, 18:1n−9	rs102275	11	C	T	0.33	0.230	0.019	2.19 × 10^−32^	−0.070	0.018	1.00 × 10^−4^
LA, 18:2n6	rs10740118	10	G	C	0.56	0.248	0.043	8.08 × 10^−9^	0.065	0.017	2.00 × 10^−4^
	rs174547	11	C	T	0.32	1.474	0.042	4.98 × 10^−274^	−0.070	0.018	1.00 × 10^−4^
	rs16966952	16	G	A	0.69	0.351	0.044	1.23 × 10^−15^	−0.023	0.019	0.223
AA, 20:4n6	rs174547	11	T	C	0.68	1.691	0.025	3.00 × 10^−971^	0.070	0.018	1.00 × 10^−4^
	rs16966952	16	G	A	0.69	0.199	0.031	2.43 × 10^−10^	−0.023	0.019	0.223
ALA, 18:3n3	rs174547	11	C	T	0.33	0.016	0.001	3.47 × 10^−64^	−0.070	0.018	1.00 × 10^−4^
EPA, 20:5n3	rs3798713	6	C	G	0.43	0.035	0.005	1.93 × 10^−12^	0.014	0.017	0.434
	rs174538	11	G	A	0.72	0.083	0.005	5.37 × 10^−58^	0.066	0.019	4.00 × 10^−4^
DPA, 22:5n3	rs780094	2	T	C	0.41	0.017	0.003	9.04 × 10^−9^	0.044	0.018	0.012
	rs3734398	6	C	T	0.43	0.040	0.003	9.61 × 10^−44^	0.011	0.017	0.516
	rs174547	11	T	C	0.67	0.075	0.003	3.79 × 10^−154^	0.070	0.018	1.00 × 10^−4^
DHA, 22:6n3	rs2236212	6	G	C	0.57	0.113	0.014	1.26 × 10^−15^	−0.010	0.017	0.571

Abbreviations: Chr = Chromosome; EAF = Effect allele frequency; SE = Standard error; SNP = Single-nucleotide polymorphism; PA = Palmitic acid; SA = Stearic acid; POA = Palmitoleic acid; OA = Oleic acid; LA = Linoleic acid; AA = Arachidonic acid; ALA = α-Linolenic acid; EPA = Eicosapentaenoic acid; DPA = Docosapentaenoic acid; DHA = Docosahexaenoic acid. ^a^ Summary statistics for PUFA from fatty acids “Circ Cardiovasc Genet 2013;6(2):171–183. doi:10.1161/CIRCGENETICS.112.964619.”, “PLoS Genet 2011;7(7):e1002193. doi:10.1371/journal.pgen.1002193” and “Circ Cardiovasc Genet 2014;7(3):321–331. doi:10.1161/CIRCGENETICS.113.000208”. ^b^ Summary statistics estimated by multiple linear regression model for frailty index from UK biobank data.

**Table 2:** Summary statistics for sensitivity analysis using proxy SNPs to consider the possibility of palindromic SNPs in three traits of fatty acids.

			Effect Allele	Other Allele	Fatty Acids ^a^				Frailty Index ^b^		
Traits	SNPs	Chr			EAF	*β*	SE	*p*	*β*	SE	*p*
SA, 18:0	rs6675668	1	G	T	0.51	0.165	0.019	2.16 × 10^−18^	0.016	0.017	0.360
	rs1803468 ^c^	1	G	A	0.88	0.170	0.029	3.681 × 10^−9^	−0.012	0.028	0.666
	rs102275	11	T	C	0.68	0.180	0.019	1.33 × 10^−20^	0.070	0.018	1.00 × 10^−4^
LA, 18:2n6	rs10761741 ^c^	10	G	T	0.56	0.251	0.044	8.77 × 10^−9^	0.066	0.019	4.00 × 10^−4^
	rs174547	11	C	T	0.32	1.474	0.042	4.98 × 10^−274^	−0.070	0.018	1.00 × 10^−4^
	rs16966952	16	G	A	0.69	0.351	0.044	1.23 × 10^−15^	−0.023	0.019	0.223
EPA, 20:5n3	rs4713165 ^c^	6	C	T	0.42	0.037	0.006	4.84 × 10^−11^	0.006	0.019	0.753
	rs174538	11	G	A	0.72	0.083	0.005	5.37 × 10^−58^	0.066	0.019	4.00 × 10^−4^

Abbreviations: Chr = Chromosome; EAF = Effect allele frequency; SE = Standard error; SNP = Single-nucleotide polymorphism; PA = Palmitic acid; SA = Stearic acid; POA = Palmitoleic acid; OA = Oleic acid; LA = Linoleic acid; AA = Arachidonic acid; ALA = α-Linolenic acid; EPA = Eicosapentaenoic acid; DPA = Docosapentaenoic acid; DHA = Docosahexaenoic acid. ^a^ Summary statistics for PUFA from fatty acids “Circ Cardiovasc Genet 2013;6(2):171–183. doi:10.1161/CIRCGENETICS.112.964619.”, “PLoS Genet 2011;7(7):e1002193. doi:10.1371/journal.pgen.1002193” and “Circ Cardiovasc Genet 2014;7(3):321–331. doi:10.1161/CIRCGENETICS.113.000208”. ^b^ Summary statistics estimated by multiple linear regression model for frailty index from UK biobank data. ^c^ Summary statistics for frailty index from UK biobank data identified by the LDproxy.

**Table 3:** MR results of the fatty acids and frailty index.

			MR			Multivariate MR ^a^		
			*β*	(95%CI)	*p*	*β*	(95%CI)	*p*
**Non-PUFAs**								
	**Saturated fatty acids**							
		Palmitic acid (16:0)	−0.063	(−0.255, 0.129)	0.518	0.288	(0.128, 0.447)	<0.001
		Stearic acid (18:0)	0.178	(0.050, 0.307)	0.007	0.361	(0.155, 0.567)	0.001
	**Mono-unsaturated fatty acids**							
		Palmitoleic acid (16:1n-7)	−1.127	(−1.868, −0.387)	0.003	0.026	(−1.083, 1.135)	0.963
		Oleic acid (18:1n-9)	−0.304	(−0.458, −0.150)	<0.001	−0.086	(−0.330, 0.158)	0.488
**PUFAs**								
	**n-6 PUFAs**							
		Linoleic acid (18:2n6)	−0.039	(−0.063, −0.016)	0.001	1.075	(−1.549, 3.698)	0.422
		Arachidonic acid (20:4n6)	0.039	(0.018, 0.060)	<0.001	−0.266	(−0.937, 0.406)	0.438
	**n-3 PUFAs**							
		α-Linolenic acid (18:3n3)	−4.379	(−6.615, −2.143)	<0.001	−44.36	(−186.03, 97.32)	0.539
		Eicosapentaenoic acid (20:5n3)	0.722	(0.323, 1.122)	<0.001	−7.865	(−50.28, 34.55)	0.716
		Docosapentaenoic acid (22:5n3)	0.849	(0.442, 1.255)	<0.001	23.09	(−51.07, 97.25)	0.542
		Docosahexaenoic acid (22:6n3)	−0.088	(−0.390, 0.215)	0.571	4.482	(−5.569, 14.533)	0.382

Abbreviations: MR = Mendelian randomization with inverse variance weighted method using a fixed-effect model; 95% CI = 95% confidence interval; Multivariate MR = Multivariable Mendelian randomization. PUFA = polyunsaturated fatty acids. ^a^ Multivariate model for non-PUFAs included palmitic acid, stearic acid, palmitoleic acid, and oleic acid as exposure variables. Multivariate model for PUFAs included linoleic acid, arachidonic acid, α-Linolenic acid, eicosapentaenoic acid, docosapentaenoic acid, and docosahexaenoic acid as exposure variables.

**Table 4:** Sensitivity analysis: MR results of the fatty acids and frailty index using the proxy SNPs.

	MR			Multivariate MR ^a^		
	*β*	(95%CI)	*p*	*β*	(95%CI)	*p*
**Stearic acid (18:0)**	0.177	(0.049, 0.305)	0.007	0.360	(0.154, 0.566)	0.001
**Linoleic acid (18:2n6)**	−0.039	(−0.062, −0.016)	0.001	−0.046	(−0.927, 0.835)	0.919
**Eicosapentaenoic acid (20:5n3)**	0.658	(0.263, 1.054)	0.001	9.832	(−3.754, 23.417)	0.156

Abbreviations: MR = Mendelian randomization with inverse variance weighted method using a fixed-effect model; 95% CI = 95% confidence interval; Multivariate MR = Multivariable Mendelian randomization. PUFA = polyunsaturated fatty acids. ^a^ Models were the same as the multivariate MR in Table 3.

## Data Availability

This research has been conducted using the UK Biobank Resource under Application Number 22224. The analyses on UK Biobank genotypes were enabled by resources provided by the Swedish National Infrastructure for Computing (SNIC) at Uppmax partially funded by the Swedish Research Council through grant agreement no. 2018-05973. The data underlying the results presented in the study are available from the UK Biobank (http://biobank.ndph.ox.ac.uk/showcase/ [accecced on 1 October 2021]).
